# Identification of potential biomarkers for diagnosis of pancreatic and biliary tract cancers by sequencing of serum microRNAs

**DOI:** 10.1186/s12920-019-0521-8

**Published:** 2019-05-16

**Authors:** Kwondo Kim, DongAhn Yoo, Hee Seung Lee, Kyong Joo Lee, Soo Been Park, Chanyang Kim, Jung Hyun Jo, Dawoon E. Jung, Si Young Song

**Affiliations:** 1 Interdisciplinary Program in Bioinformatics, Seoul National University, Seoul, Republic of Korea; 2 C&K genomics, C-1008, H businesspark, 26, Beobwon-ro 9-gil, Songpa-gu, Seoul, Republic of Korea; 3 Division of Gastroenterology, Department of Internal Medicine, Yonsei University College of Medicine, 50-1 Yonsei-ro, Seodaemun-gu, Seoul, Republic of Korea; 4 Division of Gastroenterology, Department of Internal Medicine, Hallym University Hangang Sacred Heart Hospital, Hallym University College of Medicine, Seoul, Republic of Korea; 5 Institute of Gastroenterology, Department of Internal Medicine, Yonsei University College of Medicine, 50-1 Yonsei-ro, Seodaemun-gu, Seoul, Republic of Korea

**Keywords:** Serum miRNA, Pancreatic cancer, Biliary tract cancer, Diagnosis, Biomarker

## Abstract

**Background:**

Pancreatic and biliary tract cancer (PC and BTC, respectively) are difficult to diagnose because of their clinical characteristics; however, recent studies suggest that serum microRNAs (miRNAs) might be the key to developing more efficient diagnostic methods for these cancers.

**Methods:**

We analysed the genome-wide expression of serum miRNAs in PC and BTC patients to identify novel biomarker candidates using high-throughput sequencing and experimentally validated miRNAs on clinical samples.

**Results:**

Statistical and classification analysis of the serum miRNA-expression profiles of 55 patient samples showed distinguishable patterns between cancer patients and healthy controls; however, we were unable to distinguish the two cancers. We found that three of the highest performing miRNAs were capable of distinguishing cancer patients from controls, with an accuracy of 92.7%. Additionally, dysregulation of these three cancer-specific miRNAs was demonstrated in an independent sample group by quantitative reverse transcription polymerase chain reaction.

**Conclusions:**

These results suggested three candidate serum miRNAs (mir-744-5p, mir-409-3p, and mir-128-3p) as potential biomarkers for PC and BTC diagnosis.

**Electronic supplementary material:**

The online version of this article (10.1186/s12920-019-0521-8) contains supplementary material, which is available to authorized users.

## Background

Pancreatic and biliary tract cancer (PC and BTC) are associated with high mortality rates, with reported survival rates for PC barely exceeding 17% in the United States [1] while those for cholangiocarcinoma patients at advanced unresectable stage and with gallbladder cancer are < 5% [2] and < 13% [3], respectively. The high fatality rate has triggered extensive research on these cancers; however, there has not been remarkable progress in PC and BTC diagnosis. Diagnosis of these cancers is complex due to the lack of symptoms and/or the difficulty of performing direct and invasive methods because of the anatomical positions of the pancreas and biliary tract. Additionally, widely used non-invasive diagnostic methods, including imaging technologies (computed tomography, magnetic resonance imaging, and endoscopic ultrasound) and biomarkers [serum carbohydrate antigen (i.e., CA 19–9)], are limited by their low sensitivity or specificity [4–7]. Therefore, developing better diagnostic markers for PC and BTC represents an important clinical issue.

To overcome limitations associated with current diagnostic methods, studies have focused on the development of reliable biomarkers [8–11], including noncoding RNAs, such as microRNA (miRNA), which are typically 22 nucleotides long and capable of binding to specific recognition sites on mRNAs. By silencing or reducing the expression of ~ 60% of genes in the human genome [12], miRNAs alter the activities of tumour suppressors or key regulators associated with cancer [13]. Although the exact pathways involving many of these miRNAs are not fully understood, miRNA dysregulation is frequently observed in different types of cancers, resulting in excessive cell proliferation, inhibition of apoptosis, and abnormal cellular migration [14–18].

Some miRNAs localize within cell mass, whereas others are found outside of a cell and in circulating blood, thereby designating them as serum miRNAs, which are stable and resistant to RNase attack, unlike the majority of RNAs found within cells [19]. Additionally, serum miRNAs can be easily sampled using non-invasive methods, making them promising biomarker candidates. Although the detailed function of serum miRNAs is even less-understood than other miRNAs, numerous studies predict that these miRNAs represent an efficient biomarker for the diagnosis of cancers [20–22].

MiRNAs can be identified using next-generation sequencing technology. In particular, RNA sequencing enables rapid and sensitive quantification of miRNA profiles present in the human genome. Extremely low or high expression levels can be detected using this method relative to microarray analysis, increasing the reliability of RNA-specific studies [23]. Therefore, cancer studies have increasingly focused on the development of miRNA biomarkers by employing sequencing-based quantification [24–26].

In this study, we investigated the profiles of serum miRNAs derived from PC and BTC patients and compared these levels with those of healthy controls (HCs) in order to discover candidate biomarkers for PC and BTC classification. Sequence reads of serum miRNAs were generated using high-throughput sequencing, and their expression levels were profiled by quantifying the sequence reads. Statistical and classification analyses were employed to profile and detect significantly dysregulated serum miRNAs between the groups, which were finally validated in independent sample groups.

## Results

### Differentially expressed miRNAs between three sample groups

**Fig. 1:**
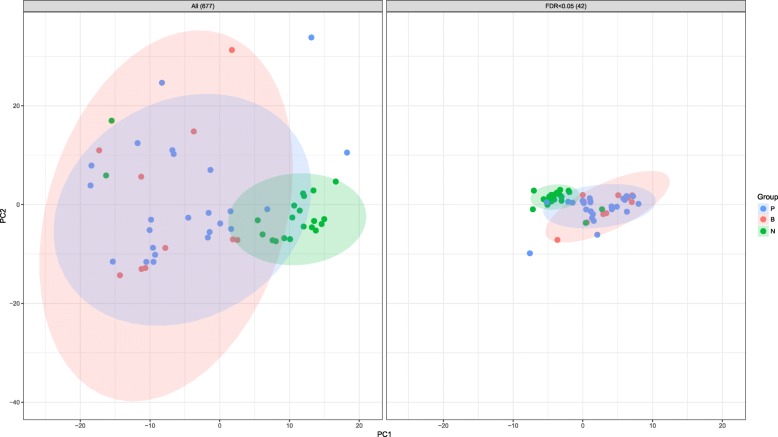
PCA evaluation of differential serum miRNA expression**.** PCA for miRNA expression in the three sample groups (677 miRNAs and 42 differentially expressed miRNAs; FDR-adjusted *p* ≤ 0.05)

After alignment of miRNA sequence data against the human miRNA database (miRBase v21; http://www.mirbase.org/), 677 miRNAs were detected in blood samples. Subsequent principal component analysis (PCA) visualized sample distribution in a two-dimensional scatter plot without using information concerning the designated group of individual samples, revealing separate clusters between the cancer and HC groups. However, PCA analysis was unable to distinguish PC and BTC individuals (Fig. 1). Additionally, the optimal number of clusters was estimated at two according to silhouette scoring using two types of correlation coefficients (Additional file 1: Figure S1). These data indicated that the overall miRNA-expression pattern was distinguished according to the presence of cancer.

Statistical analysis of the 677 miRNAs was performed to identify differentially expressed miRNAs potentially capable of distinguishing the three groups (PC, BTC, and HC). After multiple regression analysis and adjusting for clinical covariates, including age, gender, and body mass index (BMI), 42 candidate miRNAs differentially expressed in one of the three groups were identified [false discovery rate (FDR)-adjusted *p* ≤ 0.05]. PCA was then performed on the reduced set of miRNAs (Fig. 1), resulting in closely distributed samples between groups. Additionally, PCA of the 42 differentially expressed miRNAs separated most of the cancer patients from the HCs; however, distribution of PC and BTC samples remained nearly identical. PCA was also performed on a different subset of miRNAs (*p* ≤ 0.01 and 0.001). The result of PCA demonstrated that the differentially expressed miRNAs were effective for distinguishing cancer patients from HCs but were ineffective at distinguishing between the two cancers (Additional file 1: Figure S2).

**Fig. 2:**
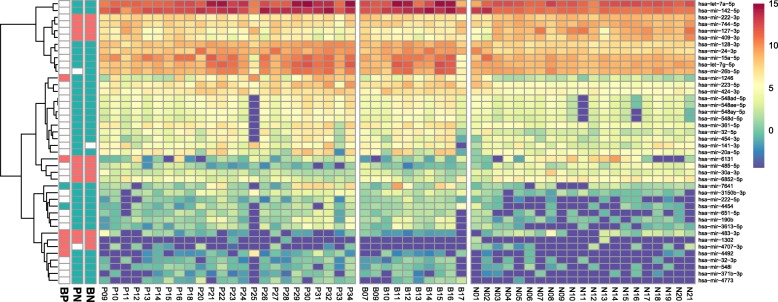
Serum miRNA expression in 55 samples. The leftmost three bar plots indicate the significant fold changes (> 2 fold) in miRNA expression among the three pairwise comparisons (BP: BTC vs. PC; PN: PC vs. HC; and BN: BTC vs. HC). The directionality of the fold change was presented by green (up) and red (down) colours

Visualization of the expression levels of the 42 miRNAs from each sample (Fig. 2) showed clearly distinguishable patterns between cancer and HC groups, except for a few outliers, including two individuals of the HC group (N01 and N02) who were diagnosed with intrahepatic and gallbladder stones. Similar to PCA results, miRNA-expression patterns in PC and BTC patients did not show distinct patterns. Additionally, pairwise comparisons according to the fold change in each of the 42 miRNAs were conducted between the three groups (Fig. 2). Although the majority of the miRNAs displayed similar expression levels between the PC and BTC groups, eight miRNAs showed fold changes > 2 (Additional file 1: Figure S3 and S4).

### Efficacy of differentially expressed miRNAs as potential biomarkers

**Fig. 3:**
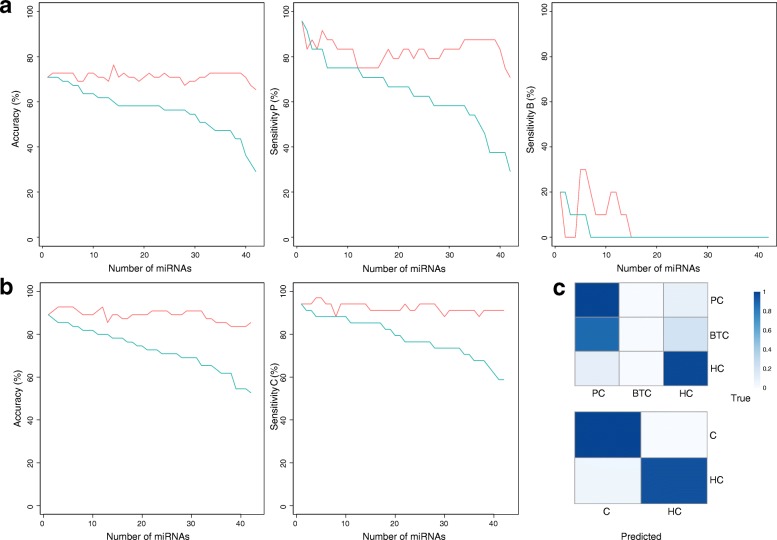
Classification accuracy and sensitivity**.** Classification accuracy and sensitivity for classifying (**a**) three groups (PC, BTC, and HC) and (**b**) two groups (cancer and non-cancer). Green line indicates accuracy/sensitivity for each miRNA, and the red line shows cumulative accuracy/sensitivity when each miRNA was added to the prediction model in descending order of accuracy/sensitivity. (**c**) Contingency table of classification results for the highest accuracy. The proportion of samples falling into the predicted group (column) and the true group (row) is represented by colour intensity (blue)

To assess the efficacy of the 42 differentially expressed miRNAs for cancer diagnosis, we evaluated their performance as potential biomarkers, and selected an optimal subset of miRNAs for PC and BTC detection. The optimal accuracy in classification of the three groups (PC, BTC, and HC) was 76.4%. This value could not be improved by using additional miRNAs, which resulted in fluctuating cumulative-accuracy values (Fig. 3a). The highest sensitivity of > 90% was observed for PC; however, this classification could only detect ≤30% of BTC patients. The cumulative sensitivity of BTC dropped to 0% upon the addition of more miRNAs for classification, thereby interfering with the distinct miRNA patterns specifically associated with BTC. This signified the high similarity between the miRNA signatures of PC and BTC, as additional miRNAs used for analysis resulted in higher incidences of BTC being mistaken for PC, generating false positives (Fig. 3c). The candidate biomarkers for classification of PC and BTC, including the eight miRNAs exhibiting fold changes > 2 (Additional file 1: Figure S3), also failed to distinguish the two cancers during three-group classification (Additional file 1: Table S1). However, when classification was conducted between the cancer and HC groups, the overall performance of classification improved. The highest accuracy of 92.7% was achieved for this two-group classification. Use of only the four miRNAs that derived the best sensitivity, 97.1% of the cancer patients were accurately detected (Fig. 3b).

In terms of accuracy, the miRNA with the highest performance in two-group classification was hsa-mir-142-5p (89.1%), followed by hsa-mir-128-3p (87.3%), hsa-mir-222-3p (85.5%), hsa-mir-6852-5p (85.5%), and hsa-mir-744-5p (85.5%) (Additional file 1: Table S2). Among these five miRNAs, the highest sensitivity (91.2%) was achieved by hsa-mir-222-3p and hsa-mir-6852-5p, followed by hsa-mir-142-5p (88.2%), hsa-mir-744-5p (88.2%), and hsa-mir-128-3p (85.3%). However, in terms of specificity, hsa-mir-142-5p and hsa-mir-128-3p performed better (90.5%) than hsa-mir-744-5p (81.0%), hsa-mir-222-3p (76.2%), and hsa-mir-6852-5p (76.2%). Overall, the highest cumulative accuracy of 92.7% was achieved when the first three of the best performing miRNAs (hsa-mir-142-5p, hsa-mir-128-3p, and hsa-mir-222-3p) were used for classification. The highest cumulative accuracy was maintained until the use of six miRNAs, which resulted in fluctuating results up to 12 miRNAs, followed by a gradual decrease in accuracy as more miRNAs were used for classification (Fig. 3b).

Classification analysis indicated that the performance of miRNAs as biomarkers was far more effective for classification between the cancer and HC groups as compared to three-group classification. The decreased accuracy in three-group classification was due to the lack of specificity in distinguishing between the two cancers (Fig. 3c). Although PC patients were predicted correctly and with high sensitivity, the majority of BTC patients were incorrectly predicted as PC, resulting in decreased overall accuracy and BTC sensitivity (Fig. 3c).

### Functional annotation of candidate-biomarker targets

To infer the biological function of the selected miRNAs, we investigated their potential involvement in different biological processes. Functional annotation was performed on the list of genes known to be regulated by the 42 differentially expressed miRNAs. The clustering results indicated high enrichment in biological process related to transcription-regulatory mechanisms, apoptotic processes, and cell proliferation (Table 1). Additionally, Kyoto Encyclopaedia of Genes and Genomes (KEGG) analysis identified a number of pathways directly related to cancer. Moreover, of the 65 genes associated with pathways related to “Pancreatic cancer”, 34 were regulated by the 42 miRNAs (Table 2). These results suggested that the identified miRNAs interact with genes closely related to cancer or cancer-related biological processes and implies that these miRNAs might represent potential biomarkers for PC and BTC diagnosis.

**Table 1:** Biological processes (Gene Ontology terms) associated with the 42 differentially expressed miRNAs

Biological process	Count	%	*P*-value	Fold Enrichment	Bonferroni
transcription, DNA-templated	581	13.30	4.86E-13	1.29	3.52E-09
positive regulation of transcription, DNA-templated	188	4.30	4.20E-12	1.58	3.04E-08
regulation of transcription, DNA-templated	448	10.25	2.78E-10	1.29	2.01E-06
negative regulation of transcription from RNA polymerase II promoter	238	5.45	4.90E-10	1.43	3.54E-06
positive regulation of transcription from RNA polymerase II promoter	304	6.96	4.36E-09	1.34	3.16E-05
cellular response to hypoxia	48	1.10	2.53E-08	2.17	1.83E-04
negative regulation of transcription, DNA-templated	168	3.84	5.34E-08	1.46	3.86E-04
positive regulation of cell migration	76	1.74	6.07E-08	1.79	4.39E-04
transforming growth factor beta receptor signalling pathway	45	1.03	1.66E-07	2.12	0.001199
apoptotic process	181	4.14	9.54E-07	1.38	0.00688
peptidyl-serine phosphorylation	54	1.24	1.21E-06	1.87	0.008702
positive regulation of cell proliferation	151	3.46	3.27E-06	1.40	0.02335
protein autophosphorylation	67	1.53	4.51E-06	1.69	0.03212
G1/S transition of mitotic cell cycle	45	1.03	5.50E-06	1.91	0.03899

Gene Ontology designations were generated via DAVID functional annotation. Only terms with a Bonferroni-adjusted p ≤ 0.05 are presented

**Table 2:** KEGG pathways of the 42 differentially expressed miRNAs

KEGG Pathway	Count	%	P-value	Fold Enrichment	Bonferroni
Pathways in cancer	158	3.62	1.19E-13	1.69	3.49E-11
FoxO signalling pathway	68	1.56	2.26E-11	2.14	6.65E-09
Glioma	40	0.92	3.67E-10	2.59	1.08E-07
Chronic myeloid leukaemia	42	0.96	1.22E-09	2.46	3.58E-07
Prostate cancer	47	1.08	5.20E-09	2.25	1.53E-06
Neurotrophin signalling pathway	56	1.28	7.08E-08	1.97	2.08E-05
Cell cycle	57	1.30	1.01E-07	1.94	2.97E-05
ErbB signalling pathway	44	1.01	1.37E-07	2.13	4.03E-05
Hepatitis B	63	1.44	2.44E-07	1.83	7.16E-05
p53 signalling pathway	36	0.82	3.64E-07	2.27	1.07E-04
PI3K-Akt signalling pathway	122	2.79	6.84E-07	1.49	2.01E-04
Viral carcinogenesis	80	1.83	1.08E-06	1.65	3.16E-04
Non-small cell lung cancer	31	0.71	1.24E-06	2.33	3.64E-04
Bladder cancer	25	0.57	1.76E-06	2.57	5.17E-04
Renal cell carcinoma	34	0.78	1.82E-06	2.21	5.35E-04
Pancreatic cancer	34	0.78	1.82E-06	2.21	5.35E-04
Proteoglycans in cancer	77	1.76	3.21E-06	1.62	9.43E-04
TGF-beta signalling pathway	40	0.92	3.82E-06	2.01	0.001123
MAPK signalling pathway	93	2.13	4.08E-06	1.54	0.001198
Signalling pathways regulating pluripotency of stem cells	58	1.33	4.85E-06	1.75	0.001425
Fc epsilon RI signalling pathway	34	0.78	6.49E-06	2.11	0.001905
Small cell lung cancer	39	0.89	1.52E-05	1.93	0.004457
Ras signalling pathway	82	1.88	2.03E-05	1.53	0.00595
Melanoma	34	0.78	2.05E-05	2.02	0.005999
T cell receptor signalling pathway	44	1.01	3.41E-05	1.80	0.009978
Endocytosis	90	2.06	4.31E-05	1.47	0.01259
Epstein-Barr virus infection	70	1.60	5.12E-05	1.55	0.014945
Hippo signalling pathway	58	1.33	6.68E-05	1.62	0.019459
Insulin signalling pathway	54	1.24	7.01E-05	1.65	0.020398
Thyroid hormone signalling pathway	46	1.05	1.15E-04	1.70	0.033145
Oestrogen signalling pathway	41	0.94	1.50E-04	1.75	0.043177
Acute myeloid leukaemia	27	0.62	1.56E-04	2.03	0.044742
Rap1 signalling pathway	74	1.69	1.58E-04	1.49	0.045381
Colorectal cancer	29	0.66	1.59E-04	1.97	0.045785

KEGG pathways were generated by DAVID functional annotation. Only terms with a Bonferroni-adjusted p ≤ 0.05 are presented

### Quantitative reverse transcription polymerase chain reaction (qRT-PCR) validation of the candidate biomarkers

**Fig. 4:**
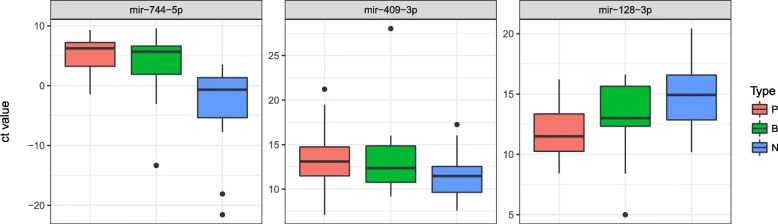
Box plot demonstrating the expression of three miRNAs validated by qRT-PCR**.** Red, green, and blue colours represent PC, BTC, and HC groups, respectively

Validation was conducted on the miRNAs displaying high performance (> 80% accuracy). MiRNA-expression levels were re-examined against an independent sample group (Additional file 1: Table S3) using qRT-PCR and a pairwise *t* test between the cancer and control groups. Results showed that two miRNAs, mir-128-3p and mir-409-3p, were significantly dysregulated in the cancer group as compared with the HC group (*p* = 2.85E− 9 and *p* = 0.0405, respectively). Additionally, mir-744-5p, with a *p*-value slightly higher than 0.05, was identified (*p* = 0.0562) (Fig. 4). The combination of the three serum miRNAs showed 87.3% accuracy and 91.2% sensitivity in classification analysis.

## Discussion

The lack of symptoms combined with inefficient diagnostic methods pose a challenge for detecting PC and BTC. Even direct diagnostic methods involving invasive procedures, such as endoscopic ultrasonography guided fine-needle aspiration biopsy, are not effective due to the difficulty of performing the method and its low sensitivity [27, 28]. Additionally, it is commonly accepted that more than cytological evidence is needed for reliable diagnosis. Therefore, diagnosis of these cancers can benefit from the use of efficient biomarkers, of which serum miRNAs are considered attractive potential candidates. Strengths of their use include inexpensive cost and convenient sampling; therefore, in response to rising demands for cancer biomarkers, numerous studies have attempted to detect serum miRNA expression in various cancer types, including PC and BTC. Chen et al. [29] identified serum miRNAs biomarker candidates for lung and colorectal cancers, whereas Mar-Aguilar et al. [30] suggested that serum miRNA profiles were capable of distinguishing breast cancer patients from HCs with high sensitivity and specificity. These findings suggested that serum miRNAs are promising biomarkers for cancers.

In this study, the expression profiles of serum miRNAs were compared with those of normal individuals in order to identify novel biomarkers for PC and BTC. Our results showed that the PC and BTC groups could not be distinguished according to serum miRNA profiles. A possible explanation is the shared biological processes between PC and BTC, which would result in similar miRNA-expression patterns. Another possible reason concerns differences in the clinical conditions of each patient. Although we attempted to minimize these differences by adjusting for clinical covariates, including age, gender, and BMI, other clinical information, including cancer stage, was not addressed. Such differences can result in noise, making it difficult to distinguish PC and BTC. Some of the patients diagnosed with stage IV PC and BTC also represent a problem for classification. Because cancer cells at this stage spread to other tissues, miRNA profiles might be altered, resulting in indistinguishable patterns. Therefore, we concluded that the current data were unable to distinguish between cancer groups. Similar miRNA-expression profiles between PC and BTC patients were also reported previously [29].

We then focused on classifying the two groups (the cancer groups and HCs). Compared to three-group classification (PC, BTC, and HC), two-group classification exhibited improved performance in classification; however, the presence of outlier miRNA-expression profiles (N01 and N02) decreased accuracy and sensitivity. Specifically, these outliers in the HC group, who showed miRNA profiles similar to those of PC and BTC patients, were diagnosed with intrahepatic and gallbladder stones, leading to false-positive results during classification. This might suggest an association between gallstone disease and cancers, agreeing with previous studies reporting that the risk of BTC and PC increases 2-fold in patients with gallstones [30, 31]. Moreover, a positive correlation between gallstone volume and the risk of gallbladder cancer was also reported [32]. Similar diseases related to PC or BTC were also found to complicate PC and BTC diagnosis [27, 33]. These findings suggest that PC and BTC might be closely related to stone-related diseases. The inability to distinguish such conditions from PC or BTC represents a limitation for the use of the serum miRNA identified in this study as potential biomarkers. However, our findings also indicated that classification performance using serum miRNAs might be improved in the absence of outlier consideration through the incorporation of a prescreening step specific for stone-related diseases.

Early diagnosis of PC and BTC is difficult due to a lack of symptoms, as well as the anatomical positions of the organs. This leads to high mortality rates. Therefore, biomarkers that can detect early stages of PC and BTC can be more effective in improving the survival rate of patients. However, we included all stages of PC and BTC in our analysis, as the difficulty in early diagnosis and sampling resulted in a lack of samples with early stage disease. In addition, we did not account for stage information in the differential expression analysis. The expression of miRNAs fluctuates throughout stage progression. Thus, accounting for stage information is generally preferable, as this may increase the total number of candidate markers by identifying differential expression across different stages. However, in our case, the reliability of markers identified in stages with extremely small sample sizes needs to be considered. We thus used the all of the PC and BTC samples as a factor instead of adjusting for stage information. In addition, the expression of serum miRNAs among different stages was analysed using PCA and heatmaps (Additional file 1: Figure S5 and Figure S6), which did not show distinctive patterns according to different stages. Using this method, only strong signals of miRNAs that can distinguish these cancers from control subjects, regardless of stage, can be detected. Since the aim of our study was to identify a small number of efficient markers with strong signals that can distinguish cancers, we believe that application of our model without adjusting for stage information is a more suitable approach, although the usage of the candidate markers cannot be confined to the diagnosis of early-stage PC and BTC.

Given the distinguishing pattern of miRNA expression between the cancer and HC groups, it is possible that dysregulated miRNAs play roles in pathways associated with cancer. Indeed, this argument was supported by the results of functional annotation analysis (Table 1), which revealed that the cluster of genes regulated by the dysregulated miRNAs were significantly enriched in biological pathways associated with cancer. Based on this observation, we investigated the potential function of each of the three miRNAs validated in this study in cancer-related pathways.

Few studies have focused on the association of miR-744 with PC and BTC, with one study reporting its overexpression in a tumour cell isolated from a PC patient and resulting in its role promoting tumorigenicity by repressing negative regulators of the Wnt/β-catenin-signalling pathway [34]. Another study reported overexpression of plasma miR-744 and suggested its potential as a diagnostic and prognostic biomarker for PC [35]. However, in the present study, we observed significant downregulation of serum mir-744-5p, which is the primary form of miR-744. The same observation was confirmed in a validation experiment using an independent dataset. Although the precise reason for this difference in findings could not be ascertained, it is predicted that the discrepancy in this miRNA-expression pattern might result from other layers of negative regulation.

MiR-409-3p is implicated in various types of cancer, with tissue miR-409-3p levels downregulated in bladder cancer, lung adenocarcinoma, gastric cancer, and breast cancer, and circulating miR-409-3p levels also downregulated in prostate cancer [34, 36–39]. In prostate cancer, circulating miR-409-3p functions as repressor of metastasis, with this miRNA binding to the 3′ untranslated region of the pro-metastatic gene radixin to suppress its expression. A previous study also reported that miR-409-3p downregulation is associated with metastasis [37]. Similar these previous findings, we observed downregulation of mir-409-3p in the PC and BTC groups in our study, supporting its reported role as a tumour suppressor in PC and BTC.

A previous study showed downregulation of tissue miR-128-3p in hepatocellular carcinoma, suggesting that miR-128-3p suppresses cancer by repressing the expression of phosphoinositide 3-kinase (PI3K), which is key to the PI3K/AKT-signalling pathway [40]. However, in other cancers, including acute lymphoblastic leukaemia and gastric cancer, miR-128-3p is upregulated [41, 42], functioning as a negative regulator of the tumour-suppressor gene plant homeodomain finger 6 in leukaemia specifically, and supporting its various roles in different cancers. In the present study, we observed that serum miR-128-3p was upregulated in the PC and BTC groups, suggesting its oncogenic role in these cancers.

In summary, our findings identified three serum miRNAs (mir-744-5p, mir-409-3p, and mir-128-3p) dysregulated in various types of cancer, including PC and BTC; however, the expression patterns of these miRNAs varied between cancer types. Although further studies are required to explain the inconsistencies observed in these expression patterns, we suggest these serum miRNAs as potential biomarkers for PC and BTC based on their distinct expression patterns relative to the HC group in our study.

## Conclusions

In this study, we profiled serum miRNA expression in samples derived from PC and BTC patients and HCs. Serum miRNA-expression profiles failed to distinguish between the two types of cancer; however, statistical and classification analyses revealed three serum miRNAs (mir-744-5p, mir-409-3p, and mir-128-3p) as effective for discriminating PC and BTC. Although tissue or circulatory levels of the three miRNAs have been suggested as representing biomarkers for PC or other cancers, our findings suggested that serum miRNAs can be also useful for PC and BTC detection.

## Methods

### Sample information and miRNA-seq experiments

A summary of information concerning the 55 samples is presented in Table 3. Serum miRNA-expression levels were quantified for each sample, including those for 24 PC patients, 10 BTC patients, and 21 HCs. Note that two of the HCs (N01 and N02) were diagnosed as having intrahepatic and gallbladder stones. The average age of the HCs (43.9 years) was lower than that of the PC and BTC patients (mean ages: 62.75 and 62.8 years, respectively). The proportion of males in the PC group (54.2%) was higher than that of females, whereas this was not the case in the BTC (30% males) and HC (28.5% males) groups.

**Table 3:** Summary of sample information

	Pancreatic cancer (*n* = 24)	Biliary tract cancer (*n* = 10)	Healthy control (*n* = 21)
Age, year, mean ± SD	62.8 ± 11.1	62.8 ± 7.8	43.9 ± 11.8
Sex (%)			
Male	11 (45.8%)	7 (70.0%)	15 (71.4%)
Female	13 (54.2%)	3 (30.0%)	6 (28.6%)
Diabetes (%)	8 (33.3%)	5 (50.0%)	1 (4.8%)
Hypertension (%)	12 (50.0%)	3 (30.0%)	1 (4.8%)
Smoking (%)	4 (16.7%)	5 (50%)	0 (0.0%)
BMI, kg/m^2^, mean ± SD	21.7 ± 2.9	23.2 ± 3.9	23.8 ± 3.8
CA19–9, U/ml, mean ± SD	3444.5 ± 6543.2	807.1 ± 1457.9	7.9 ± 6.9
Tumor size, mm, mean ± SD	31.2 ± 11.1	34.2 ± 26.5	n/a
Stage (%)			
I	2 (8.3%)	2 (20.0%)	n/a
II	9 (37.5%)	3 (60.0%)	n/a
III	0 (0.0%)	1 (10.0%)	n/a
IV	13 (54.2%)	4 (40.0%)	n/a
Recurrence after surgery (%)	4/11 (36.4%)	7/8 (87.5%)	n/a
DFS, median (range)	10.5 (3.1–46.8)	15.9 (2.1–44.1)	n/a
OS, month, median (range)	15.2 (3.7–56.8)	21.7 (2.8–44.7)	n/a

Abbreviations: SD, standard deviation; BMI, body mass index; DFS, disease-free survival; OS, overall survival

Serum samples were collected in 10-mL BD serum tubes and centrifuged at 4 °C for 20 min at 3000 rpm. The supernatant was then aliquoted, and total RNA containing miRNA was extracted from the samples using the serum miRNA purification kit (Genolution, Seoul, Korea) according to manufacturer instructions. Libraries were prepared for 50-bp single-end sequencing using the NEXTflex small RNA-seq kit (Bioo Scientific, Austin, TX, USA). Small RNA molecules were isolated from 1 μg of total RNA via adapter ligation, followed by synthesis as single-stranded cDNAs through reverse-transcription priming. By applying these products as a template for second-strand synthesis, double-stranded cDNA was prepared by PCR, and fragments (~ 150 bp) were extracted for sequencing according to size selection following gel electrophoresis. The quality of the cDNA libraries was evaluated using the Agilent 2100 BioAnalyzer (Agilent Technologies, Santa Clara, CA, USA), followed by quantification with the KAPA library quantification kit (Kapa Biosystems, Wilmington, MA, USA) according to manufacturer protocol. Following cluster amplification of the denatured templates, single-end (50 bp) sequencing progressed using an Illumina HiSeq2500 system (Illumina, San Diego, CA, USA).

### miRNA-seq data pre-processing and expression quantification

Quality control was performed on raw sequence data using fastQC-0.11.3 [43], followed by the deletion of potential adapter and low-quality sequences using Trimmomatic-0.32 [44] prior to sequence alignment. Trimmed reads with lengths not within ~ 16–35 bp were filtered out. Reads were aligned against miRBase version 21 [45] and quantified using miRDeep2 [46]. Unique matches with miRNA sequences were quantified, allowing one mismatch. MiRNAs expressed (> 10 reads) in at least two samples were retrieved.

### Clustering analysis according to serum miRNA expression profiles

To investigate relationships between samples, we employed PCA using different miRNA subsets, and silhouette score [47] was used to estimate the optimal number of clusters. We used the cluster package implemented in R to calculate the silhouette score [48] using hierarchical clustering, with Pearson and Spearman correlation coefficients as distance measures.

### Statistical analysis associated with detection of differentially expressed miRNAs

The expression levels of 677 miRNAs were normalized using the trimmed mean of M-values method [49] implemented in edgeR to account for sequence depth for each samples [50]. For each normalized miRNA-expression value, a statistical test was performed to identify differentially expressed miRNAs between different groups (PC, BTC, and HC) while adjusting for covariates using edgeR [50]. Among the patient information available in our data, age, gender, and BMI were selected as covariates [51–53]. Stage information (I–IV) was not considered due to the small subgroup size of each cancer stage, which could potentially lead to misleading results (i.e. reduced statistical power and reliability of the analysis due to small sample size). The model focused on the cancer group as a whole, rather than focusing on individual stages. Given the null hypothesis that effects of the group were zero, the significance of statistical testing for each miRNA-expression value was calculated using the likelihood ratio test and adjusted by the Benjamini and Hochberg method [54] to control for multiple testing errors.

### Classification analysis to test the performance of potential biomarkers

The K-nearest neighbour (KNN) algorithm, a representative heuristic method, classifies an instance according to a majority vote of its k nearest neighbours [55]. Several studies have successfully employed this algorithm for cancer classification based on miRNA expression [56–58]. The KNN algorithm was used here to select miRNAs and classify patients with different health statuses according to a Euclidean distance metric between miRNA-expression values.

Choosing an optimal k value for the KNN classifier is a critical step in improving the performance of the classification model. Optimal nearest neighbour of K = 11 was selected in this study based on the proportion of majority votes and accuracies generated by bootstrapping (Additional file 1: Figure S7). The performance of the classification model constructed by the given set of miRNAs was evaluated by leave-one-out cross-validation.

### Functional annotation of candidate-miRNA-target genes

To infer the biological function of candidate miRNAs, functional annotation was performed on the list of genes known to be regulated by the miRNAs using DAVID [59]. The experimentally curated miRNA-target gene interactions were retrieved from miRTarBase version 7.0 [60].

### qRT-PCR validation of detected miRNAs

Reverse transcription and qRT-PCR were performed using a TaqMan Advanced miRNA cDNA synthesis kit (Applied Biosystems, Foster City, CA, USA), TaqMan Advanced miRNA assays (Applied Biosystems), and TaqMan Fast Advanced master mix (Applied Biosystems) according to manufacturer protocols. qRT-PCR was performed using an ABI Prism 7300 system (Applied Biosystems), and primers for mature miRNAs were purchased from Applied Biosystems. PCR amplification consisted of an initiation step at 95 °C for 10 min, followed by 45 cycles at 95 °C for 30 s, 56 °C for 30 s, and 72 °C for 15 s. All qRT-PCR assays were performed in triplicate using total RNA samples from 17 PC patients, 17 BTC patients, and 19 HCs. To identify dysregulated miRNAs, a pairwise *t* test was performed to compare the miRNA-expression levels of cancer and HC groups.

## Additional file

Additional file1: **Figure S1.** Optimal cluster estimation based on silhouette score. **Figure S2.** Principal component analysis using different miRNA subsets. **Figure S3.** Box plot of miRNA expression for PC (P), BTC (B), and HC (N) groups. **Figure S4.** Volcano plot of miRNAs. **Figure S5.** Principal component analysis of serum miRNA expression according to stage. **Figure S6.** Serum miRNA expression according to stage. **Figure S7.** Parameter optimization of the K-nearest neighbour algorithm. **Table S1.** Three-group classification performance by the miRNAs. **Table S2.** Two-group classification performance by the miRNAs. **Table S3.** Summary of validation sample information. (DOCX 967 kb)

## Abbreviations

BMI: body mass index
BTC: biliary tract cancer
DFS: disease-free survival
FDR: false discovery rate
HC: healthy control
KEGG: Kyoto Encyclopaedia of Genes and Genomes
KNN: K-nearest neighbour
miRNA: microRNA
OS: overall survival
PC: pancreatic cancer
PCA: principal component analysis
PI3K: phosphoinositide 3-kinase
qRT-PCR: quantitative reverse transcription polymerase chain reaction
SD: standard deviation
