# Supplementary material for: Identification of potential biomarkers for diagnosis of pancreatic and biliary tract cancers by sequencing of serum microRNAs
Source: BMC Med Genomics. 2019 May 16;12:62. doi: 10.1186/s12920-019-0521-8 (PMC6524273; doi:10.1186/s12920-019-0521-8)
Supplement: Supplementary file 1 — Figure S1. Optimal cluster estimation based on silhouette score. Figure S2. Principal component analysis using different miRNA subsets. Figure S3. Box plot of miRNA expression for PC (P), BTC (B), and HC (N) groups. Figure S4. Volcano plot of miRNAs. Figure S5. Principal component analysis of serum miRNA expression according to stage. Figure S6. Serum miRNA expression according to stage. Figure S7. Parameter optimization of the K-nearest neighbour algorithm. Table S1. Three-group classification performance by the miRNAs. Table S2. Two-group classification performance by the miRNAs. Table S3. Summary of validation sample information. (DOCX 967 kb) [file 12920_2019_521_MOESM1_ESM.docx]

**Supplementary Information**

**Identification of potential biomarkers for diagnosis of pancreatic and biliary tract cancers by high-throughput sequencing of serum microRNAs**

Kwondo Kim^¶^, DongAhn Yoo^¶^, Hee Seung Lee, Kyong Joo Lee, Soo Been Park, Chanyang Kim, Jung Hyun Jo, Dawoon E. Jung^§^ and Si Young Song^§^

**
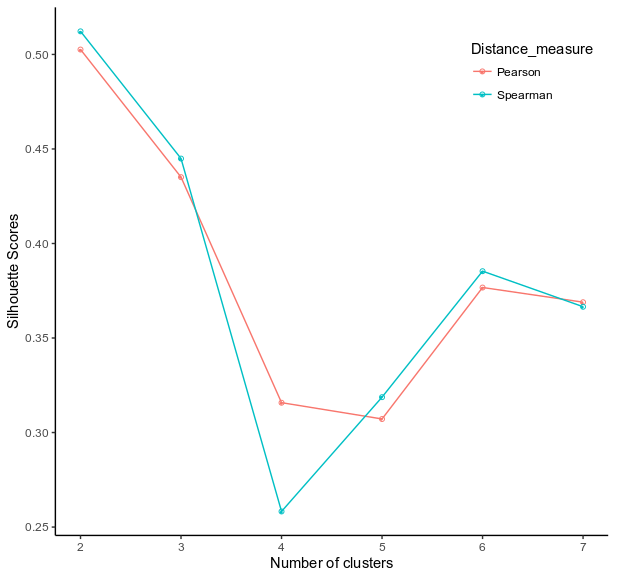
**

**Supplementary Fig. S1. Optimal cluster estimation based on silhouette score.** Silhouette score estimated using two types of correlation coefficients (Pearson and Spearman; denoted by red and blue lines, respectively). The Y-axis represents the silhouette score, and the X-axis represents the number of clusters.

**
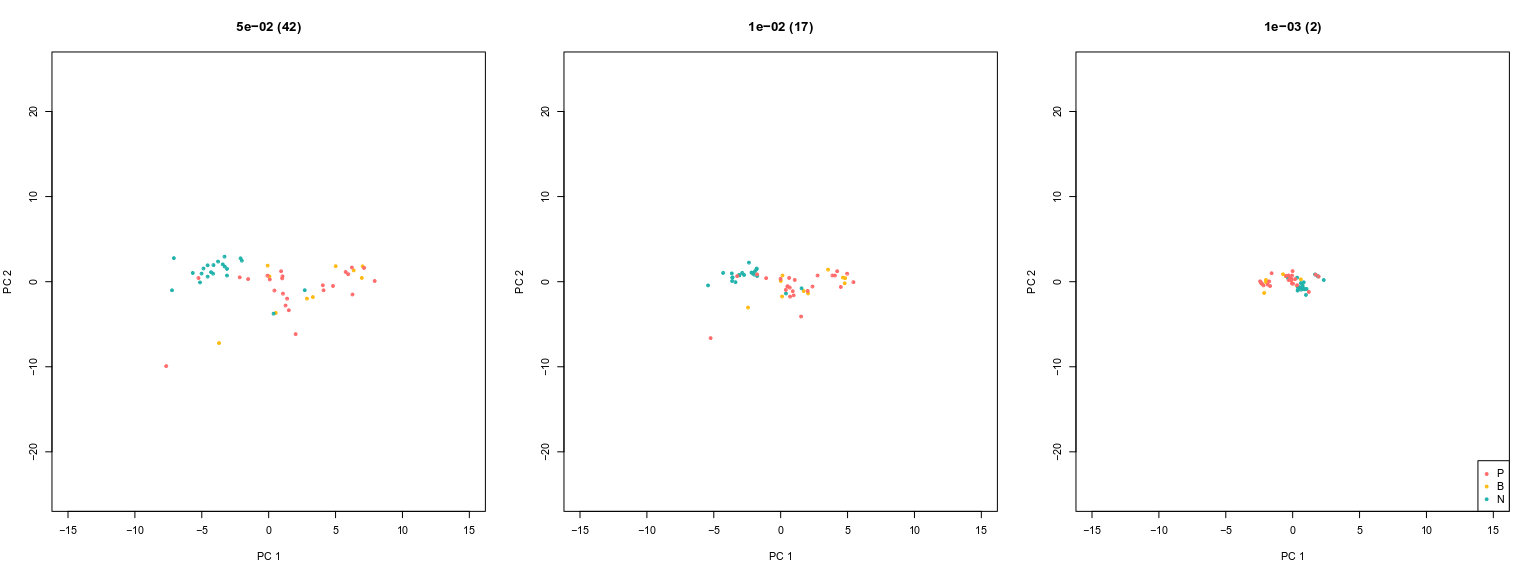
Supplementary Fig. S2. Principal component analysis using different miRNA subsets.** MiRNA subsets were selected based on different cut-off values for their adjusted p-value according to multiple regression analysis. The cut-off value is shown on top of each scatter plot, with the number in the bracket representing the number of miRNAs used.

**
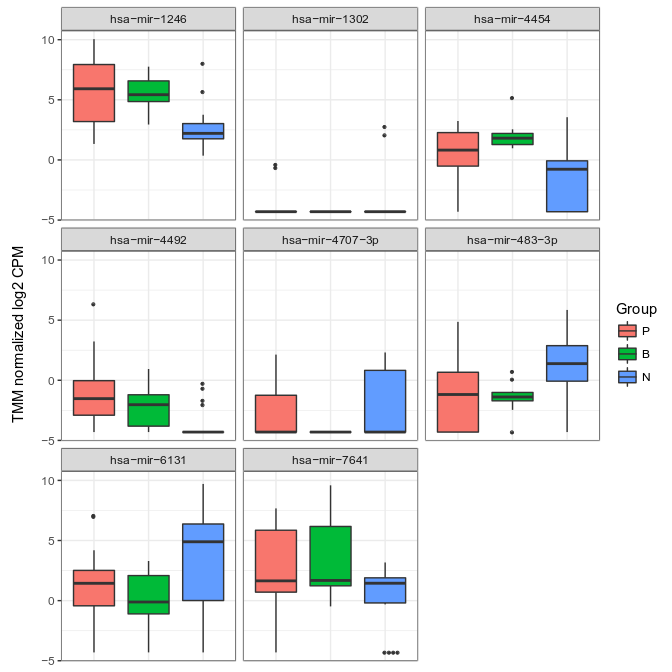
**

**Supplementary Fig. S3. Box plot of miRNA expression for PC (P), BTC (B), and HC (N) groups.** Among the 42 differentially expressed miRNAs, eight exhibited fold changes >2. The name of each miRNA is shown.

**
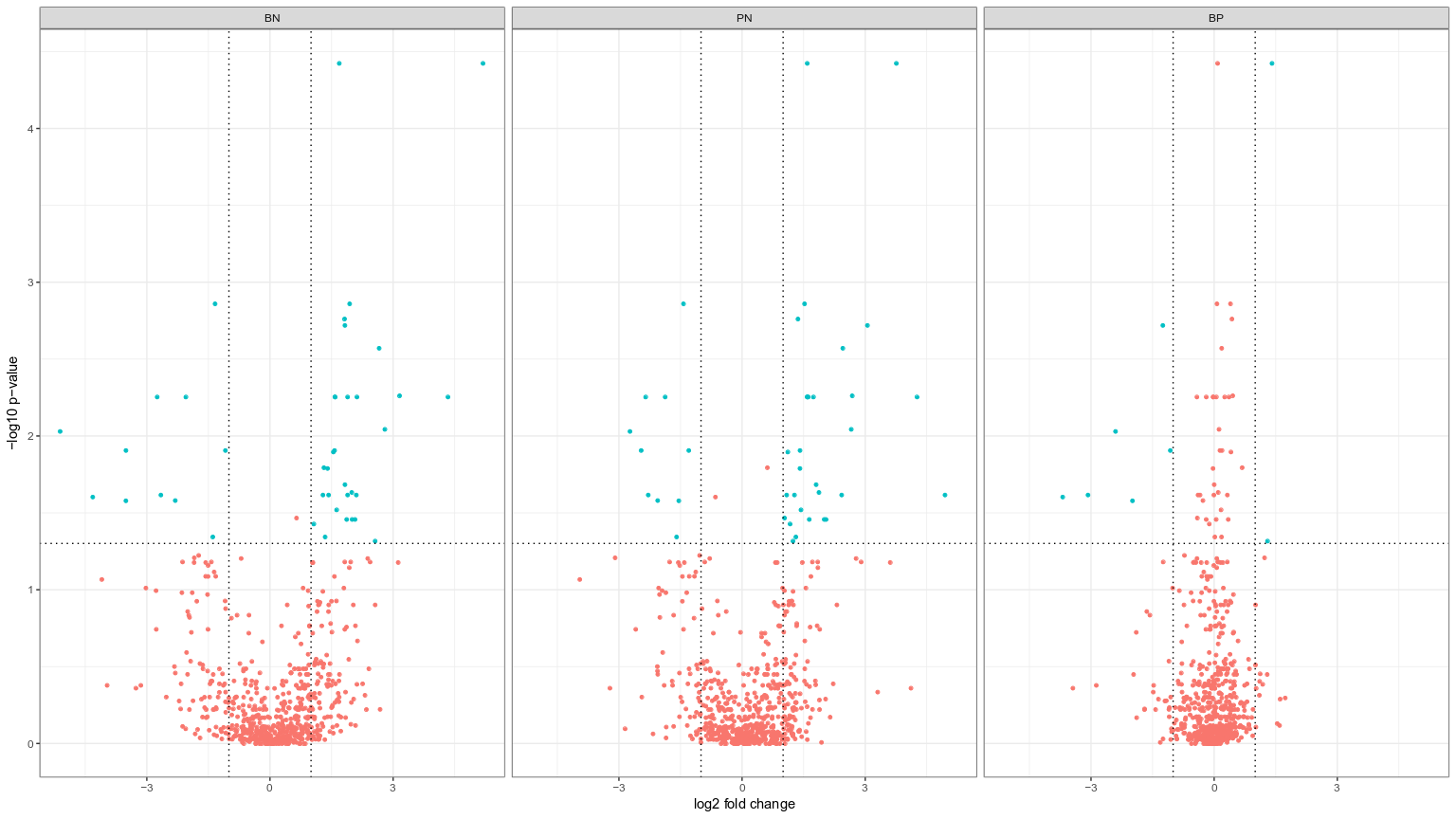
**

**Supplementary Fig. S4. Volcano plot of miRNAs.** Differential expression is shown by the log of the adjusted p-value on the Y-axis. The direction of differential expression is shown according to log_2_ fold change. Note that a cut-off of 0.05 for the adjusted p-value and +1 and −1 for the log_2_ fold change are shown as dotted lines. The blue dots represent differentially expressed miRNAs with fold changes >2.

**
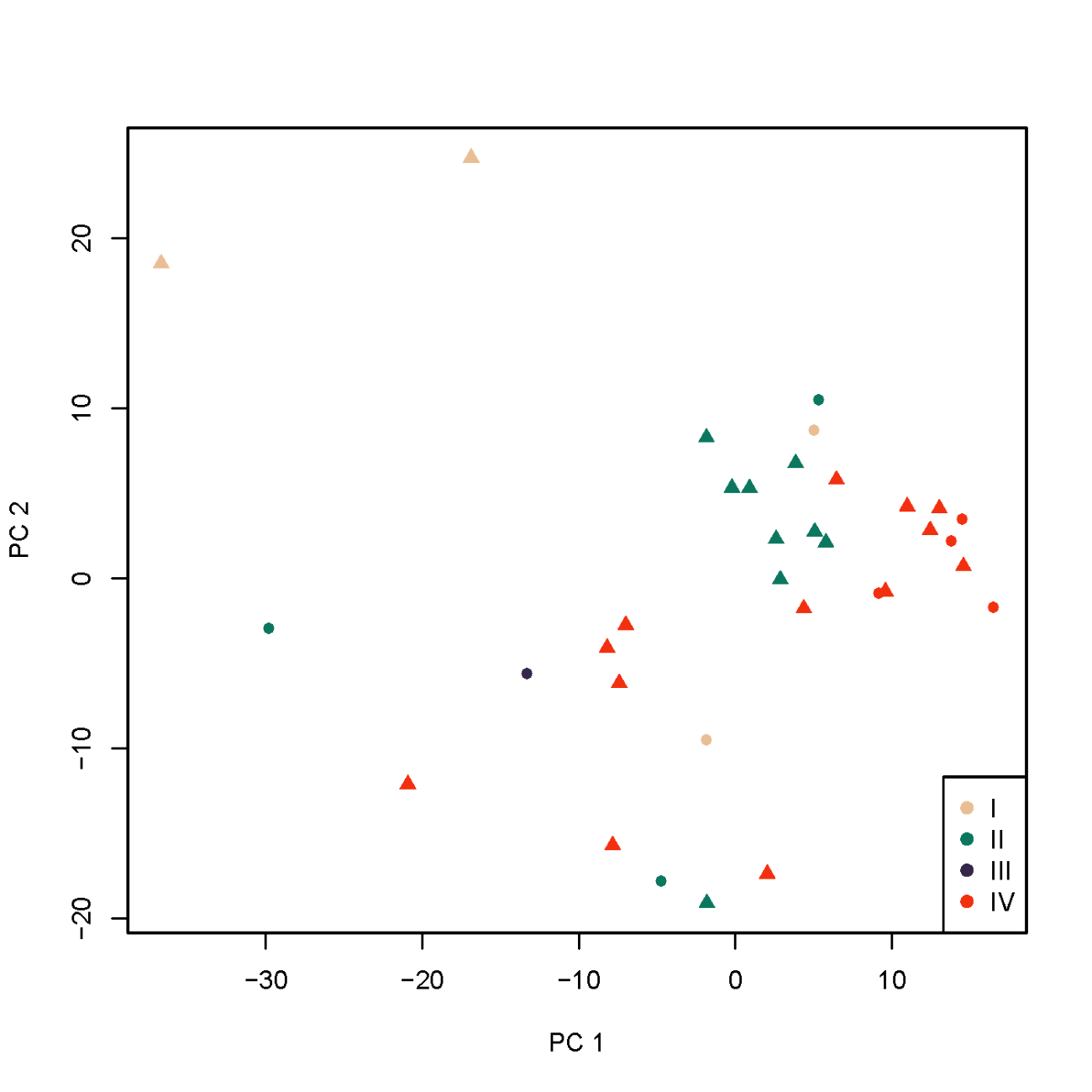
**

**Supplementary Fig. S5. Principal component analysis of serum miRNA expression according to stage.** Triangles and circles indicate PC and BTC samples, respectively.

**
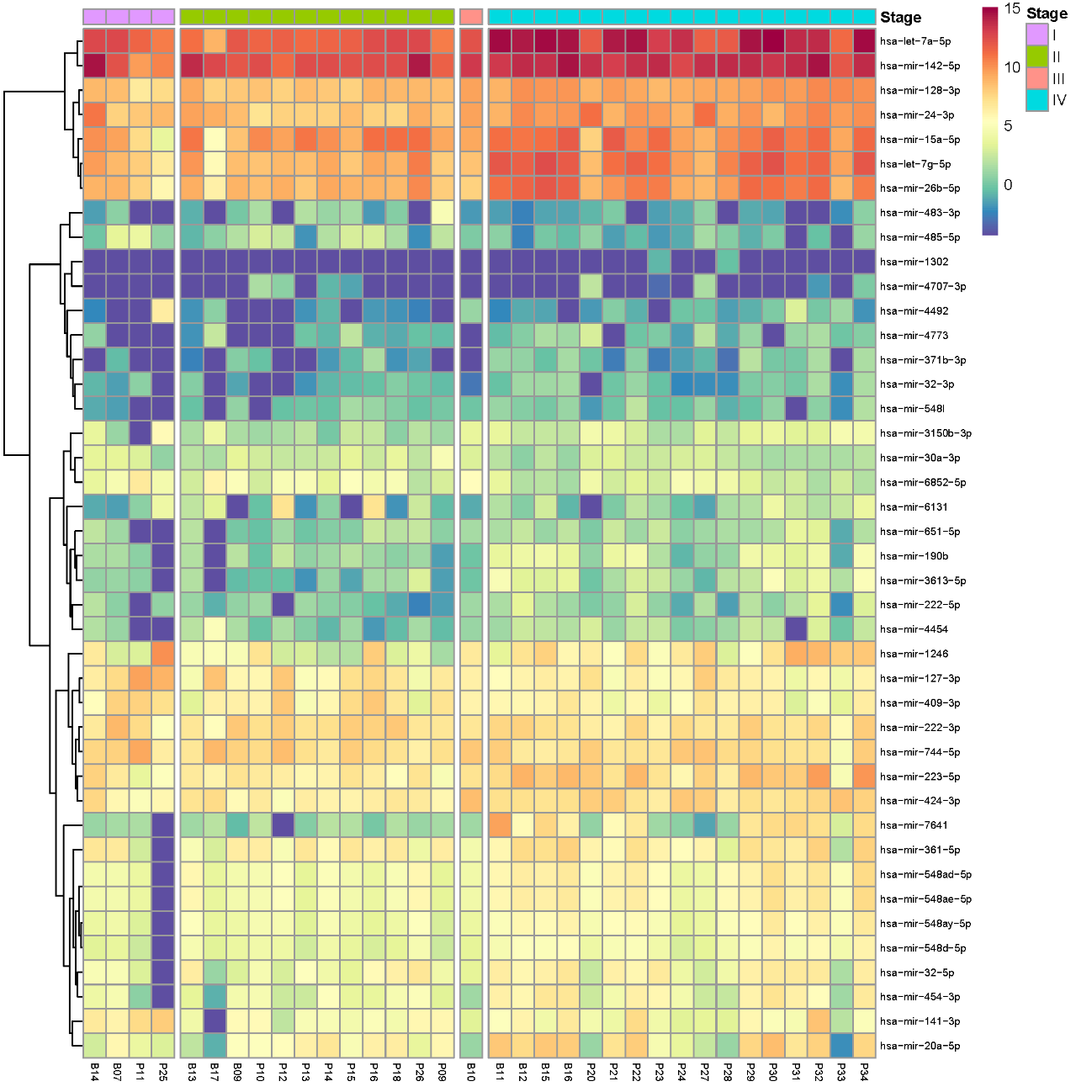
**

**Supplementary Fig. S6. Serum miRNA expression according to stage.** Stage information is presented in the first row, indicated by different colours.

**
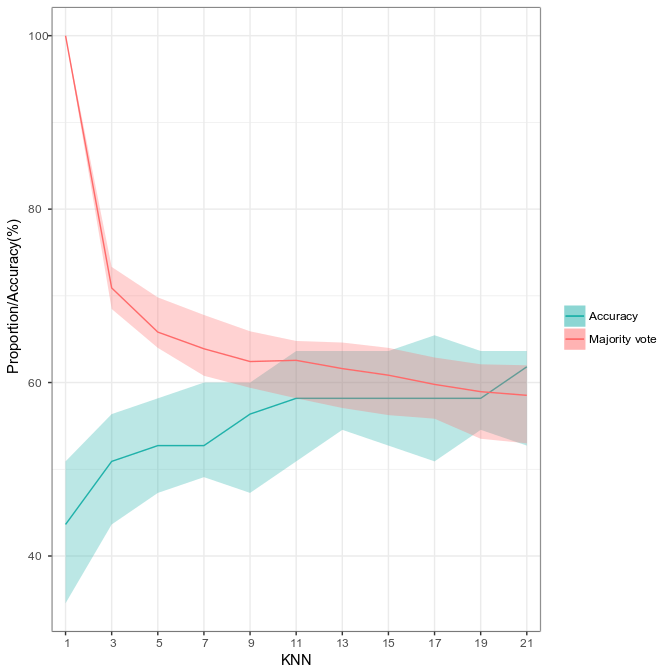
**

**Supplementary Fig. S7. Parameter optimization of the K-nearest neighbour algorithm.** Accuracy and majority vote corresponding to each K value were calculated to identify the optimal K value.

**Supplementary Table S1. Three-group classification performance by the miRNAs.** MiRNAs in red exhibited fold changes >2 between the PC and BTC groups.

| **miRNA** | **Accuracy** | **P_sensitivity** | **P_specificity** | **B_sensitivity** | **B_specificity** | **N_sensitivity** | **N_specificity** |
| --- | --- | --- | --- | --- | --- | --- | --- |
| mir-128-3p | 70.9% | 83.3% | 64.5% | 0.0% | 100.0% | 90.5% | 85.3% |
| mir-142-5p | 70.9% | 83.3% | 61.3% | 0.0% | 100.0% | 90.5% | 88.2% |
| mir-222-3p | 70.9% | 95.8% | 58.1% | 0.0% | 100.0% | 76.2% | 91.2% |
| mir-361-5p | 69.1% | 75.0% | 74.2% | 0.0% | 100.0% | 95.2% | 73.5% |
| mir-6852-5p | 69.1% | 91.7% | 58.1% | 0.0% | 100.0% | 76.2% | 88.2% |
| mir-3150b-3p | 67.3% | 83.3% | 58.1% | 0.0% | 100.0% | 81.0% | 85.3% |
| mir-4773 | 67.3% | 75.0% | 71.0% | 0.0% | 100.0% | 90.5% | 73.5% |
| let-7g-5p | 63.6% | 58.3% | 80.6% | 0.0% | 100.0% | 100.0% | 58.8% |
| mir-4492 | 63.6% | 75.0% | 64.5% | 0.0% | 100.0% | 81.0% | 73.5% |
| mir-744-5p | 63.6% | 70.8% | 67.7% | 0.0% | 88.9% | 85.7% | 85.3% |
| mir-15a-5p | 61.8% | 62.5% | 74.2% | 0.0% | 100.0% | 90.5% | 61.8% |
| mir-223-5p | 61.8% | 70.8% | 54.8% | 0.0% | 97.8% | 81.0% | 82.4% |
| mir-424-3p | 61.8% | 66.7% | 64.5% | 20.0% | 95.6% | 76.2% | 76.5% |
| mir-30a-3p | 60.0% | 66.7% | 67.7% | 0.0% | 100.0% | 81.0% | 64.7% |
| mir-127-3p | 58.2% | 66.7% | 54.8% | 0.0% | 91.1% | 76.2% | 85.3% |
| mir-20a-5p | 58.2% | 58.3% | 64.5% | 0.0% | 100.0% | 85.7% | 64.7% |
| mir-24-3p | 58.2% | 66.7% | 51.6% | 0.0% | 95.6% | 76.2% | 82.4% |
| mir-32-3p | 58.2% | 70.8% | 51.6% | 0.0% | 100.0% | 71.4% | 76.5% |
| mir-32-5p | 58.2% | 58.3% | 67.7% | 0.0% | 100.0% | 85.7% | 61.8% |
| mir-3613-5p | 58.2% | 70.8% | 61.3% | 0.0% | 100.0% | 71.4% | 67.6% |
| mir-371b-3p | 58.2% | 70.8% | 58.1% | 0.0% | 100.0% | 71.4% | 70.6% |
| mir-409-3p | 58.2% | 66.7% | 54.8% | 0.0% | 91.1% | 76.2% | 85.3% |
| mir-548d-5p | 58.2% | 54.2% | 77.4% | 0.0% | 100.0% | 90.5% | 52.9% |
| let-7a-5p | 56.4% | 75.0% | 54.8% | 0.0% | 100.0% | 61.9% | 70.6% |
| mir-1246 | 56.4% | 62.5% | 54.8% | 10.0% | 95.6% | 71.4% | 76.5% |
| mir-454-3p | 56.4% | 62.5% | 64.5% | 0.0% | 100.0% | 76.2% | 61.8% |
| mir-485-5p | 56.4% | 62.5% | 54.8% | 10.0% | 95.6% | 71.4% | 76.5% |
| mir-548l | 56.4% | 75.0% | 51.6% | 0.0% | 97.8% | 61.9% | 76.5% |
| mir-6131 | 54.5% | 75.0% | 38.7% | 0.0% | 95.6% | 57.1% | 88.2% |
| mir-651-5p | 54.5% | 75.0% | 45.2% | 0.0% | 93.3% | 57.1% | 85.3% |
| mir-222-5p | 50.9% | 58.3% | 48.4% | 10.0% | 88.9% | 61.9% | 82.4% |
| mir-548ay-5p | 50.9% | 50.0% | 74.2% | 0.0% | 95.6% | 76.2% | 50.0% |
| mir-7641 | 49.1% | 58.3% | 54.8% | 0.0% | 93.3% | 61.9% | 67.6% |
| mir-141-3p | 47.3% | 54.2% | 51.6% | 20.0% | 88.9% | 52.4% | 73.5% |
| mir-190b | 47.3% | 45.8% | 58.1% | 0.0% | 93.3% | 71.4% | 61.8% |
| mir-548ad-5p | 47.3% | 58.3% | 58.1% | 0.0% | 91.1% | 57.1% | 64.7% |
| mir-548ae-5p | 47.3% | 58.3% | 58.1% | 0.0% | 91.1% | 57.1% | 64.7% |
| mir-26b-5p | 43.6% | 37.5% | 61.3% | 0.0% | 100.0% | 71.4% | 44.1% |
| mir-4454 | 43.6% | 37.5% | 51.6% | 0.0% | 88.9% | 71.4% | 67.6% |
| mir-4707-3p | 36.4% | 37.5% | 48.4% | 10.0% | 86.7% | 47.6% | 61.8% |
| mir-483-3p | 32.7% | 37.5% | 35.5% | 0.0% | 84.4% | 42.9% | 70.6% |
| mir-1302 | 29.1% | 29.2% | 58.1% | 0.0% | 91.1% | 42.9% | 35.3% |

**Supplementary Table S2. Two-group classification performance by the miRNAs.**

| **miRNA** | **Accuracy** | **C_sensitivity** | **C_specificity** | **N_sensitivity** | **N_specificity** |
| --- | --- | --- | --- | --- | --- |
| mir-142-5p | 89.1% | 88.2% | 90.5% | 90.5% | 88.2% |
| mir-128-3p | 87.3% | 85.3% | 90.5% | 90.5% | 85.3% |
| mir-222-3p | 85.5% | 91.2% | 76.2% | 76.2% | 91.2% |
| mir-6852-5p | 85.5% | 91.2% | 76.2% | 76.2% | 91.2% |
| mir-744-5p | 85.5% | 88.2% | 81.0% | 81.0% | 88.2% |
| mir-3150b-3p | 83.6% | 88.2% | 76.2% | 76.2% | 88.2% |
| mir-409-3p | 83.6% | 88.2% | 76.2% | 76.2% | 88.2% |
| mir-1246 | 81.8% | 88.2% | 71.4% | 71.4% | 88.2% |
| mir-127-3p | 81.8% | 85.3% | 76.2% | 76.2% | 85.3% |
| mir-361-5p | 81.8% | 73.5% | 95.2% | 95.2% | 73.5% |
| mir-223-5p | 80.0% | 85.3% | 71.4% | 71.4% | 85.3% |
| mir-32-3p | 80.0% | 85.3% | 71.4% | 71.4% | 85.3% |
| mir-6131 | 80.0% | 94.1% | 57.1% | 57.1% | 94.1% |
| mir-20a-5p | 78.2% | 76.5% | 81.0% | 81.0% | 76.5% |
| mir-4773 | 78.2% | 73.5% | 85.7% | 85.7% | 73.5% |
| mir-485-5p | 78.2% | 85.3% | 66.7% | 66.7% | 85.3% |
| mir-4492 | 76.4% | 76.5% | 76.2% | 76.2% | 76.5% |
| mir-651-5p | 76.4% | 88.2% | 57.1% | 57.1% | 88.2% |
| mir-24-3p | 74.5% | 85.3% | 57.1% | 57.1% | 85.3% |
| mir-548l | 74.5% | 85.3% | 57.1% | 57.1% | 85.3% |
| let-7a-5p | 72.7% | 79.4% | 61.9% | 61.9% | 79.4% |
| mir-222-5p | 72.7% | 82.4% | 57.1% | 57.1% | 82.4% |
| mir-32-5p | 72.7% | 70.6% | 76.2% | 76.2% | 70.6% |
| mir-371b-3p | 70.9% | 73.5% | 66.7% | 66.7% | 73.5% |
| mir-424-3p | 70.9% | 76.5% | 61.9% | 61.9% | 76.5% |
| mir-4454 | 70.9% | 70.6% | 71.4% | 71.4% | 70.6% |
| mir-483-3p | 70.9% | 88.2% | 42.9% | 42.9% | 88.2% |
| let-7g-5p | 69.1% | 58.8% | 85.7% | 85.7% | 58.8% |
| mir-141-3p | 69.1% | 79.4% | 52.4% | 52.4% | 79.4% |
| mir-15a-5p | 69.1% | 67.6% | 71.4% | 71.4% | 67.6% |
| mir-190b | 69.1% | 73.5% | 61.9% | 61.9% | 73.5% |
| mir-30a-3p | 65.5% | 67.6% | 61.9% | 61.9% | 67.6% |
| mir-3613-5p | 65.5% | 76.5% | 47.6% | 47.6% | 76.5% |
| mir-454-3p | 65.5% | 67.6% | 61.9% | 61.9% | 67.6% |
| mir-548ay-5p | 63.6% | 73.5% | 47.6% | 47.6% | 73.5% |
| mir-548ad-5p | 61.8% | 76.5% | 38.1% | 38.1% | 76.5% |
| mir-548ae-5p | 61.8% | 76.5% | 38.1% | 38.1% | 76.5% |
| mir-548d-5p | 61.8% | 61.8% | 61.9% | 61.9% | 61.8% |
| mir-1302 | 54.5% | 82.4% | 9.5% | 9.5% | 82.4% |
| mir-26b-5p | 54.5% | 58.8% | 47.6% | 47.6% | 58.8% |
| mir-7641 | 54.5% | 73.5% | 23.8% | 23.8% | 73.5% |
| mir-4707-3p | 52.7% | 64.7% | 33.3% | 33.3% | 64.7% |

**Supplementary Table S3. Summary of validation sample information.**

|  | Pancreatic cancer  (n=19) | Biliary tract cancer  (n=16) | Healthy control  (n=19) |
| --- | --- | --- | --- |
| Age, year, mean ± SD | 62.9 ±8.8 | 62.6 ±11.3 | 47.8 ±10.1 |
| Sex (%) |  |  |  |
| Male | 13 (68.4%) | 9 (56.3%) | 13 (68.4%) |
| Female | 6 (31.6%) | 7 (43.8%) | 6 (31.6%) |
| Diabetes (%) | 9 (47.4%) | 3 (18.8%) | 0 (0.0%) |
| Hypertension (%) | 11 (57.9%) | 4 (25.0%) | 0 (0.0%) |
| CA19-9, U/ml, mean ± SD | 2706.6 ±6162.1 | 1767.7 ±4999.0 | 10.0 ±5.6 |
| Stage (%) |  |  |  |
| I | 1 (5.3%) | 0 (0.0%) | n/a |
| II | 3 (15.8%) | 5 (31.3%) | n/a |
| III | 7 (36.8%) | 3 (18.8%) | n/a |
| IV | 8 (42.1%) | 8 (50.0%) | n/a |
| OS, month, median (range) | 16.1 (2.5-40.3) | 20.8 (2.2-68.5) | n/a |

Abbreviations: SD, standard deviation; BMI, body mass index; DFS, disease-free survival; OS, overall survival.
